# Immunohistochemical staining of tumor necrosis factor-α and interleukin-10 in benign and malignant ovarian neoplasms

**DOI:** 10.3892/ol.2014.2781

**Published:** 2014-12-08

**Authors:** MILLENA PRATA JAMMAL, ALLISON ARAÚJO DA SILVA, AGRIMALDO MARTINS FILHO, ELIÂNGELA DE CASTRO CÔBO, SHEILA JORGE ADAD, EDDIE FERNANDO CANDIDO MURTA, ROSEKEILA SIMÕES NOMELINI

**Affiliations:** 1 Research Institute of Oncology (IPON)/Discipline of Gynecology and Obstetrics, Federal University of Triângulo Mineiro (UFTM), 38025-440 Uberaba, MG, Brazil; 2 Department of Special Pathology, Federal University of Triângulo Mineiro (UFTM), 38025-440 Uberaba, MG, Brazil

**Keywords:** ovarian cancer, ovarian neoplasms, interleukin-10, tumor necrosis factor-α, immunohistochemical staining

## Abstract

Ovarian cancer is the ninth most common malignancy and the fifth leading cause of cancer death in women in the USA. The majority of malignant tumors of the ovary are diagnosed at an advanced stage, making it the most fatal gynecological cancer. The aim of the current study was to determine whether there are differences in immunohistochemical tissue staining of cytokine tumor necrosis factor-α (TNF-α) and interleukin-10 (IL-10) between benign tumors and malignant primary ovarian cancer. In total, 28 patients undergoing surgery for ovarian cysts were evaluated, and a diagnosis of benign neoplasm (n=14) or malignant neoplasm (n=14) was determined. An immunohistochemical study of histological sections of ovarian tumors was conducted. The results were analyzed using Fisher’s exact test, with P<0.05 indicating a statistically significant difference. Immunohistochemical staining of IL-10 was increased in malignant tumors compared with benign tumors (P=0.0128). For TNF-α, the immunohistochemical staining was more intense in malignant neoplasms, however, a statistically significant difference was not observed. These results indicate that the analysis of cytokines may be useful as a potential tissue marker of ovarian malignancy.

## Introduction

Ovarian cancer is one of the most fatal malignant gynecological neoplasms (1), with the majority of tumors having reached an advanced stage prior to diagnosis. The predominant therapeutic strategy is debulking surgery followed by chemotherapy. Despite chemotherapeutic treatment with platinum and taxane derivatives, the five-year survival rate for stages III and IV is only 5–15%, while for stages I and II, it is 80–95% (2,3).

The sensitivity and specificity of gynecological screening for ovarian neoplasms are limited (4). Ultrasound may aid in the diagnosis of ovarian tumors and differentiation between neoplastic and non-neoplastic tumors (5,6); additionally, color Doppler can increase the specificity of transvaginal ultrasound (7,8). However, the early diagnosis of ovarian cancer by ultrasound may be challenging due to its low prevalence in the general population decreases the positive predictive value of the screening test (9,10).

Large numbers of leukocytes, particularly mononuclear cells such as lymphocytes and tumor-associated macrophages (TAMs), may be identified in the tumor stroma (11) and malignant effusions (12), and are able to produce Th1 and Th2 type cytokines. A progressive shift in the production of cytokines by TAMs (from Th1 to Th2 type) may occur during tumor progression, reducing the immune response to the tumor (12).

Recent studies have demonstrated the importance of tumor necrosis factor-α (TNF-α) in promoting tumor growth and in the progression of ovarian malignancies (13,14). Additionally, Zhou *et al* assessed the expression of IL-10 in primary ovarian carcinoma, and observed that the tissue level of this cytokine was significantly higher in ovarian cancer compared with benign and normal controls (15). Furthermore, the malignant cases also showed significantly high IL-10 levels in the serum and ascitic fluid, suggesting that malignant cells can synthesize interleukin, which likely assists in the promotion and development of ovarian carcinoma. The tumor microenvironment differs between benign, malignant and non-neoplastic tumors, indicating a role of cytokines in tumor progression (16). A progressive shift in the production of cytokines may occur during tumor progression. Cytokines can stimulate cell growth and contribute to metastasis. Thus, cytokines may be useful tissue markers in determining ovarian malignancy.

The aim of the present study was to determine whether differences exist in immunohistochemical staining of cytokines, TNF-α and interleukin-10 (IL-10) between benign tumors and malignant primary ovarian tumors.

## Materials and methods

### Patients

In total, 28 patients were evaluated in the Pelvic Mass Clinic, Oncological Research Institute/Discipline of Gynecology and Obstetrics, Federal University of Triângulo Mineiro (Uberaba, Brazil), and subsequently underwent surgical treatment in accordance with pre-established criteria, following which a diagnosis of benign neoplasm (n=14) or malignant neoplasm (n=14) of the ovary was confirmed. The indication criteria for laparotomy were as follows: Anechoic cysts with a maximum diameter <7.0 cm, persistence of change for more than six months and normal tumor markers; tumor markers changed; anechoic cysts with a maximum diameter ≥7.0 cm; solid ovarian tumor, presence of intracystic vegetation, thick septa, two or more thin septa; and a color Doppler resistance index ≤0.4 (17,18).

Inclusion criteria were the postoperative diagnosis of primary ovarian malignancy or benign neoplasm by pathological analysis of paraffin-embedded tissue sections. Exclusion criteria were the presence of adnexal torsion of the pedicle, rupturing of the cyst during surgery, secondary ovarian malignancy (metastasis), chemotherapy treatment prior to surgery and recurrence.

The study was reviewed and approved by the ethics committee of the Federal University of Triângulo Mineiro. Written informed consent was obtained from each patient or a family member.

### Pathological analysis

Pathological analysis was conducted by the Federal University of Triângulo Mineiro Surgical Pathology Service. Tissue samples were embedded in paraffin and analyzed by an experienced pathologist. The pathological evaluation and staging of the cases were performed according to the criteria of the International Federation of Gynecology and Obstetrics (19).

### Immunohistochemistry

Paraffin blocks containing representative samples of tumors were selected from a review of the original slides. Specimens obtained from surgical resection were fixed in 10% formalin prior to being processed in paraffin. Hematoxylin-eosin-stained sections were reviewed by a pathologist and a representative section for each case was selected for immunohistochemical analysis.

Selected sections were deparaffinized, rehydrated, and heated in a microwave oven in 0.01 M citrate buffer (pH 6.0; Química Contemporânea, Diadema, Brazil) for 30 min. Endogenous peroxidase activity was blocked by 3% hydrogen peroxide for 10 min, followed by a wash with phosphate buffered saline. The sections were incubated overnight at 4°C with the following primary antibodies: Anti-TNF (rabbit polyclonal IgG, 100 μg/ml, 1:50 dilution, cat. no. sc-130220; Santa Cruz Biotechnology, Inc., Dallas, TX, USA) and anti-IL-10 (rabbit polyclonal IgG, 1 mg/ml, 1:600 dilution, cat. no. 250713; Abbiotec, San Diego, CA, USA). The primary antibody was then detected using avidin-biotin peroxidase detection solution (Dakocytomation labelled streptavidin biotin reagent; Dakocytomation, Glostrop, Denmark and System-horseradishperoxidase; Dako, Glostrup, Denmark) and the signal was visualized using diaminobenzidine (Dakocytomation) and Substrate Chromogen-System (Dako). Slides were counterstained with Harris’s hematoxylin, dehydrated, cleared and mounted. Positive controls from the appendix and tonsils were used. The cells were initially observed at a low magnification (×100) to assess the general distribution of the primary antibody. The samples were subsequently examined at a higher magnification (×400). The evaluation of cell staining was performed in tumor tissue. The tumor cells (exhibiting gross and evident nucleoli, and irregular chromatin) were identified and counted at the higher magnification. Immunohistochemical staining was evaluated in the cytoplasm of tumor cells. The intensity of staining of each section was evaluated subjectively by three separate observers (who were blinded to the experiment) using the following designations: 0–10% of cells stained, score 0; 11–25% of cells stained, score 1; 26–50% of cells stained, score 2; 51–100% of cells stained, score 3. Those scoring 0–1 were considered to be negative, and those scoring 2–3 were considered to be positive.

### Statistical analysis

Data were analyzed using GraphPad Instat 3.0 software. For immunohistochemical staining, the concordance between staining intensity scores for each sample was calculated according to Cohen’s κ coefficient: κ <0.4, slight concordance; κ ≥0.4 and <0.8, moderate concordance; κ ≥0.8 and <1, strong concordance; and κ =1, perfect concordance. The first κ inter-rater was between 0.8 and 1.0 of 1% (between strong and perfect concordance). All discordant cases were re-evaluated and the result was determined by consensus.

The association between staining intensity and tumor classification was evaluated using Fisher’s exact test. P<0.05 was considered to indicate a statistically significant difference.

## Results

The study evaluated 28 patients, 14 with benign and 14 with malignant neoplasms. The group with benign tumors had a mean age of 37.28±13.69 years, and mean parity of 2.14±1.70 infants. Table I shows the qualitative variables of the patients that were evaluated, as well as the tumor staging. Table II demonstrates the histopathological features of the tumors.

The immunohistochemical analysis of benign and malignant histological sections for TNF-α showed absent or weak (0–1) staining in nine (64.3%) benign tumors and in six (42.9%) malignant tumors, and moderate or strong (2–3) staining in five (35.7%) benign tumors and in eight (57.1%) malignant tumors. Regarding IL-10, there was absent or weak (0–1) staining in eight (57.1%) benign tumors and 1 (7.1%) malignant tumor, and moderate or strong (2–3) staining in six (42.9%) benign tumors and 13 (92.9%) malignant tumors. Immunohistochemical staining of IL-10 was more intense in malignant tumors compared with benign tumors (P=0.0128). Immunohistochemical staining for TNF-α was more intense in malignant neoplasms compared with benign neoplasms, but this difference was not statistically significant (P=0.4495) (Table III).

## Discussion

The cytokine TNF-α is increased in malignant ovarian tumors compared with the normal ovarian surface epithelium (20). Several studies have demonstrated an association between inflammation and ovarian tumorigenesis, suggesting that TNF-α is critical in the regulation of invasion, angiogenesis, and tumor metastasis (20,21). The biological activity of TNF-α may be modulated by its membrane surface receptors (TNF-R), which are able to bind TNF with high affinity (22,23). The regulation of TNF-Rs is critical to governing the responsiveness of tumor cells to TNF-α. High plasma levels of TNF-α and TNF-Rs correlated with tumor stage and a reduced mean survival time (14). The TNF-R also has a soluble form (sTNF-R) which acts as a specific cytokine antagonist. sTNF-R binds circulating TNF-α and inhibits its biological activity by preventing its binding to cellular receptors. High concentrations of sTNF-R can inhibit TNF and therefore represent a mechanism by which tumors may escape the destructive effects of TNF-α. Although the definite pathogenic role of sTNF-Rs remains controversial, they have been proposed as reliable markers for local production of TNF-α (24,25).

Several studies have observed elevated TNF-α concentrations in the blood of ovarian cancer patients and patients with a wide variety of tumor types (26,27). These studies have also demonstrated that TNF-α is crucial in promoting tumor growth and in the progression of ovarian malignancies (14,16,28). Nowak *et al* (29) observed the increased production of TNF-α in peripheral blood mononuclear cells in patients with malignant ovarian cancer, compared with nonmalignant cases. Furthermore, the production of high levels of IL-10 and transforming growth factor-β1 (TGF-β1) by malignant cells reduces ovarian production of interferon-γ (IFN-γ) and TNF-α, suppressing the immune response against the tumor cells. However, TNF-α is not always detectable in cancer patients, and concentrations may vary between individuals and throughout the course of the disease (25,30). Our results revealed no significant difference in tissue expression of TNF-α between malignant and benign neoplasms, which may be due to its downregulation by the activity of other cytokines, including IL-10. Further evaluation with a greater number of cases is required to provide additional insight.

IL-10 is a multifunctional cytokine, produced by Th2 lymphocytes, and may inhibit cellular immune responses. It is an inhibitor of activated macrophages, which are important in the homeostatic control of innate immune responses and cellular immunity. IL-10 may exert various effects on the immune system and is associated with angiogenesis, growth and proliferation of cancer cells, together with IL-8 (31). Studies have demonstrated that IL-10 may be involved in the progression of ovarian cancer (15,32,33). The elevation of serum norepinephrine and IL-10 levels induced by chronic stress was observed to promote the growth of ovarian carcinoma in mouse models (34). TGF-β1, vascular endothelial growth factor and IL-10 were expressed in 100, 74.69 and 54.96% of malignant tissues of epithelial ovarian cancer, respectively, suggesting that these cytokines have immunosuppressive roles (35).

Zhou *et al* (15) evaluated the expression of IL-10 in primary carcinomas of the ovary, and demonstrated that the tissue level of this cytokine was significantly higher in malignant tumors compared with benign tumors and normal controls. In addition, the malignant tumors had significantly higher serum and ascitic fluid IL-10 levels, suggesting that the malignant cells were able to synthesize this IL, and that it may contribute to the promotion and development of ovarian carcinoma. These findings are in agreement with the current study, which showed more intense labeling of the cytokine IL-10 in malignant compared with benign ovarian neoplasms.

In conclusion, cytokines may be useful tissue markers in determining ovarian malignancy. However, a great amount of further research into the immune profile of ovarian neoplasms is necessary before this approach may be refined for clinical use.

## Figures and Tables

**Table I:** Qualitative variables of patients and tumor staging.

	Malignant neoplasms (n=14)		Benign neoplasms (n=14)	
		
Variable	n	%	n	%
Hormonal status				
Menacme	6	42.86	10	71.43
Menopause	8	57.14	4	28.57
Smoking status				
Yes	9	64.28	5	35.71
No	5	35.72	9	64.29
Family history of cancer				
Yes	2	14.28	3	21.43
No	12	85.72	11	78.57
Previous history of cancer				
Yes	0	0.00	0	0.00
No	14	100.00	14	100.00
Stage				
IA	5	42.86		
IC	2	14.28		
IIIB	1	7.14		
IIIC	5	42.86		
IV	1	7.14		

**Table II:** Types of tumors determined by histopathological analysis.

Tumor type	n	%
Benign neoplasms (n=14)		
Mucinous cystadenoma	7	50.00
Serous cystadenoma	3	21.43
Benign cystic teratoma	3	21.43
Serous cistoadenofibroma	1	7.14
Malignant neoplasms (n=14)		
Borderline mucinous cystadenoma	1	7.14
Mucinous cistoadenocarcinoma	1	7.14
Papillary serous adenocarcinoma	1	7.14
Endometrioid adenocarcinoma	1	7.14
Anaplastic papillary adenocarcinoma	1	7.14
Ovarian carcinoid	1	7.14
Granulosa cell tumor associated with Brenner tumor	1	7.14
Squamous cell carcinoma teratoma	1	7.14
Granulosa cell tumor	2	14.28
Dysgerminoma	2	14.28
Serous cistoadenocarcinoma	2	14.28

**Table III:** Immunohistochemical staining of TNF-α and IL-10 in benign and malignant ovarian neoplasms.

	TNF-α staining				IL-10 staining			
		
	Score 2–3		Score 0–1		Score 2–3		Score 0–1	
				
	n	%	n	%	n	%	n	%
Benign neoplasms (n=14)	5	35.7	9	64.3	6	42.9	8	57.1
Malignant neoplasms (n=14)	8	57.1	6	42.9	13	92.9	1	7.1

P=0.4495 (TNF-α) and P=0.0128 (IL-10). TNF-α, tumor necrosis factor-α; IL-10, interleukin-10.
